# Effect of long-term heat stress on grain yield, pollen grain viability and germinability in bread wheat (*Triticum aestivum* L.) under field conditions

**DOI:** 10.1016/j.heliyon.2021.e07096

**Published:** 2021-06-04

**Authors:** J.E. Shenoda, Marwa N.M.E. Sanad, Aida A. Rizkalla, S. El-Assal, Rania T. Ali, Mona H. Hussein

**Affiliations:** a Genetic Engineering and Biotechnology Research Division, Genetics and Cytology Department, National Research Center (NRC), Egypt; b Genetics Department, Faculty of Agriculture, Cairo University, Egypt

**Keywords:** Field evaluation, Germinability, Reproductive stage, Heat susceptibility index, Yield components

## Abstract

Frequent episodes of heat threaten sustainable agriculture in Egypt. This study is an urgent call to select tolerant genotypes of heat and discover the predicted screening phenotypic parameters. Here, twenty spring wheat genotypes were exposed to heat stress under field conditions for screening heat tolerance. Stress environments were simulated by delaying the sowing date by 53 and 58 days than the normal environments for two successive seasons. Stressed plants received the highest peak of heat during the reproductive growth stage. Eight phenotypic parameters were measured to evaluate genotype tolerance. Mean performance, reduction percentage/trait, and heat susceptibility index parameters were calculated. Additionally, the pollen grain viability during spike emergence and the germinability of producing grains were investigated. Results demonstrated: (1) Highly significant differences (*P < 0.01*) between genotypes, treatments and genotypes by treatments in grain yield and other traits in both studied seasons, (2) significant reduction in all studied traits compared to the non-stress environment, (3) the overall yield reduction, based on grain yield/m^2^, was 40.17, 41.19 % in the first and second seasons, respectively, and the most tolerant genotypes were Masr2, Sids1, Giza 171 and Line 9, (4) limited impact of heat has detected on pollen grains viability and germinability, and (5) grain yield as a selection criterion for heat stress remains the most reliable yardstick.

## 1 Introduction

The world faces a gradual rise in heat frequency, predicted to increase by 1.0–1.7 °C by 2050 [1]. This increase in temperature will expose most crops to heat stress during different periods of their life cycles. Globally, wheat provides food for more than 35% of the global population [2] with a total cultivation area of 2.14 million km^2^ and global production of 761 million tons [3]. In Egypt, wheat is a highly needed crop, but due to the local weather, only spring wheat can survive the Egyptian climate. Recently, wheat production in Egypt faces threats because of heat episodes during the spring, especially with late sowing. The optimal temperatures for growing spring wheat during anthesis and grain filling stages ranged from 12 to 22 °C [4]. Thus, exceeding this puts the plant under heat stress which affects almost all stages of wheat growth from germination to maturity. However, the optimal negative impact is during the reproductive phase, due to the substantial yield losses incurred by the direct effect of heat stress on grain number and mass [4, 5, 6]. Generally, heat stress on plants is commonly associated with poor seed germination impacting the size of plant population [7], reduced water and nutrient uptake [8], increased evapotranspiration [9], decreased chlorophyll content [10], inhibited photosynthetic efficiency [11], changed hormones [12], increased spikelet sterility [13], reduced source-sink activities [14], unwanted reactive oxygen species responsible for the production of oxidative stress in plants [15] and finally decreased yield [16].

Stress intensity and duration are two important factors which count in screening plant plasticity [17]. Long and prolonged duration of heat stress cause a reduction in all agronomical characters, particularly grain yield [18, 19, 20, 21], however, it was recorded that a single-day heat stress event can cause a significant reduction in grain yield and yield components [5]. In the field, delaying the sowing date, compared to normal sowing date that mimic the optimum conditions for wheat growing under same field conditions, is still a common procedure. This aims to postpone the sowing date by up to 60 days [20]. Consequently, the wheat plants experience severe heat stress at the reproductive stage which causes a reduction in plant height, spike length, number of grains/main spike, and total grain yield [20]. A delay of 30 days from November, 21^st^ to December 21^st^ is associated with substantial losses in grain yield as compared with an early sowing [22].

Different traits or phenotypic parameters have been suggested to identify heat-tolerant wheat genotypes, meanwhile breeders still rely mainly upon evaluating grain yield and other yield attributes in response to heat stress [23]. Based on that, different tolerance indices, such as Heat Response Index (HRI) [24], Stress Tolerance Index (STI) [25], and Heat Susceptibility Index (HSI), are used as indicators of yield stability and a proxy for heat tolerance in wheat [26].

Exposure to high temperature can cause considerable morpho-physiological damage including pollen viability [27] which has an adverse effect on pollen cells and microspores resulting in male sterility [28]. Even a high temperature - above 30 °C - during floret development may cause complete sterility in wheat depending on genotypes [29]. Moreover, the grain filling process (i.e. nutrient accumulation in developing and maturing grains) is sensitive to environmental conditions and has strong effects on the quality of the final yield [30]. The heat stress during seed development significantly affects seed quality, dormancy, germination, and emergence as well as seedling establishment [31, 32]. Thus, establishing the transgenerational effects of heat stress in bread wheat is of relevance, particularly for the broader wheat breeding programs that exist globally where such information could be used to enhance future breeding strategies.

The key hypothesis of this study is that if global climate changes caused a dramatic increase in temperature, there will be an adverse impact on wheat productivity in Egypt. Thus, Egyptian wheat genotypes need to be enriched with new heat-tolerant genotypes. We aim to select the most plastic genotypes to high-temperature stress and associate the reliable detective traits for selection. To achieve the proposed aim, a collection set of 20 bread wheat genotypes including local cultivars and introgression and imported lines was evaluated under Egyptian field conditions in successive episodes of heat during the reproductive stage. A set of eight traits related to yield components was investigated in association with pollen grain viability and germinability.

## 2 Materials and methods

### 2.1 Plant materials

Twenty bread wheat (*Triticum aestivum* L.) genotypes were evaluated for two successive seasons, the 1^st^ season (S1) was during 2015/2016 and the same source of seeds used for the 2^nd^ season (S2) during 2016/2017. See Table 1 for the information regarding the studied materials.

**Table 1:** A list of the evaluated wheat genotypes including their names, pedigree and origins.

Entry	Name	Origin	Pedigree
1	Sids 1	Egypt	HD21/PAVON″S″//1158.57/MAYA74″S″
2	Sids 12	Egypt	BUC//7C/ALD/5/MAYA74/ON//1160-147/3/BB/GLL/4/CHAT“S”/6/MAYA/VUL//CMH74A.630//4∗SX
3	Sids 13	Egypt	AMAZ19 = KAUZ“S”//TSI/SNOB“S”
4	Masr 2	Egypt	SKAUZ/BAV92
5	Shandwel 1	Egypt	SITE/MO/4/NAC//3∗PVN/3/MiRLO
6	Gemmiza 9	Egypt	ALD″S″/HUAC ″S″//CMH74A 630/SX
7	Gemmiza 10	Egypt	Maya 74 “S”/On//1160-147/3/Bb/4/Chat”S”/5/ctow.
8	Gemmiza 11	Egypt	B0W"S"/KVZ"S"//7C/SERI82/3/GIZA168/SAKHA61.CGM7892-2GM—1GM-2GM–1GM0GM
9	Giza 171	Egypt	Sakha 93/Gemmiza 9 S.6-1GZ-4GZ-1GZ-2GZ-0S
10	Line 1	Egypt	(G 164 × 1228) (Kv2/Buha “s” Kal/Bb)x (134 x 5.69//86/3/386/7)
11	Line 2	Egypt	(562 x 1203)=(Local 2052 x 5500-10-21/29) 134 x 5.69–186/3/368/1
12	Line 3	Egypt	(G164 × 1204)=(Kv2/Buha “s” Kal/Bbx)
13	Line 4	CIMMYT	KINGBIRD#1//INQALAB91∗2/TUKURU
14	Line 5	CIMMYT	MILAN/KAUZ//PRINIA/3/BAV92
15	Line 6	CIMMYT	QUAIU#//MILAN/AMSEL
16	Line 7	CIMMYT	ATTILA
17	Line 8	CIMMYT	FRNCLN/3/ND643//2∗PRL/2∗PASTOR/4/FRANCOLIN#1 CMSS08Y00896T-099TOPM-099Y-099M-099NJ-24WGY-0B
18	Line 9	CIMMYT	TRCH∗2//ND643/2∗WBLL1 CMSS08B00602T-099TOPY-099M-099NJ-4WGY-0B
19	Line 10	CIMMYT	BLOUK#1/4/WHEAR/KUKUNA/3/C80.1/3∗BATAVIA//2∗WBLL1/5/. CMSS08B00633T-099TOPY-099M-099NJ-11WGY-0B
20	Line 11	CIMMYT	WHEAR//2∗PRL/2∗PASTOR/3/KIRITATI/2∗TRCH/4/WHEAR//2∗PRL/. CMSS08B00903T-099TOPY-099M-099NJ-10WGY-0B

The Egyptian genotypes from 1 to 9 were kindly provided by Agriculture Research Center, Giza, Egypt. Line1, 2, and 3 are unregistered lines developed in Assiut University, Assiut, Egypt. Line 4: Line 11 are imported lines from CYMMIT.

### 2.2 Experimental design and stress description

Wheat genotypes were grown in clay-loam soil (physiochemical properties, Sand%: 33.1, Silt%: 34.6, Clay%: 32.3; P^H^: 7.61, E. C. (ds/m): 1.61) at the Cairo University Research Farm, Giza, Egypt. The heat stress was applied by the routine procedure of delaying sowing date 53 and 58 days than the normal sowing dates at S1 and S2, respectively. The normal and heat stress environments are referred to as NE and SE, respectively. Plants were grown in plots per genotype; each plot size is 1.5 ^x^ 1.5 m^2^, with row-to-row spacing of 25cm. Surface supplementary irrigation during wheat growth period was added as needed. Fertilization and all other agricultural practices were performed as per the wheat growing protocol. Maximum and minimum temperatures were recorded periodically, according to the Egyptian Meteorological Authority (EMA).

### 2.3 Measurements and analysis

All the following data were measured in both seasons except pollen viability and germinability were evaluated for S2 only.

#### 2.3.1 Phenological, morphological and agronomical parameters

According to the Zadoks scale [33], days to heading (DTH) and days to maturity (DTM) were counted from sowing date till Z55 (Half of the ear emerged above flag leaf ligule) and Z92 (Seeds can no longer divide by thumbnail), respectively. The difference between Z55 to Z92 was counted as grain filling duration (GFD). Morphological and agronomical parameters were evaluated, following CIMMYT physiological breeding procedures guidelines [34]. These parameters included plant height (PH), spike length (SL), and spike number/m^2^ (SNO). At maturity, all plants in each plot were harvested (excluding border rows), and the grain yield/m^2^ (GY/m^2^) and 100-kernel weight (KW) were measured.

#### 2.3.2 Pollen grain viability

Three spikes per replication from each genotype in each treatment were collected at the proper stage (Z55 to Z57). Anthers were excised with a sharp needle and preserved in -20 °C until the microscopic examination was performed according to the method of [35, 36]. The number of viable and non-viable pollen grains was examined and counted.

#### 2.3.3 Germinability

Fifteen seeds per genotype in each replicate (i.e. those obtained from mixed of 30 random spikes grown in SE and NE conditions during S2) were sterilized by rinsing with 70% ethanol for 5 min, then washed with sterile distilled water. The seeds were germinated on filter paper (Whatman No. 1) in Petri dishes containing sterile distilled water for 120 h at 20 °C. The test was repeated three times for each genotype per treatment. The seed exhibiting radical emergence was scored as germinated. The germinability percentage was represented as follows:

Germinability % = number of seeds germinated within 5 days /total number of seed X100.

#### 2.3.4 Heat susceptibility index (HSI)

Heat Susceptibility Index (HSI) was calculated to the grain yield and the other traits for each season and for each genotype following the equation number (1) for [37]:

HSI = (1-Y/Yp)/D

where

Y = mean of the target trait in the stress environment per genotype (SE).

Yp = mean of the same target trait in the normal environment per genotype (NE).

D = Stress Intensity = 1–X/Xp.

X = mean of Y of all studied genotypes.

Xp = mean of Yp of all studied genotypes.

HSI was calculated to characterize the level of tolerance to heat stress, compared with all studied genotypes. In this index, the genotype is very tolerant with a value lower than 0.5 but if the value is between 0.5 and 1 the tolerance goes toward the moderate level. Any genotype scores more than 1.00 is recorded as susceptible.

### 2.4 Statistical analysis

This study was focused on studying two factors, the environments and genotypes. This is why the experiments were laid out in the field in a split-plot design with three replications where environments (treatments) were randomly assigned as main plots and genotypes were randomized as subplots within the main plots. The means and standard deviation for each trait of all studied genotypes were calculated.

Furthermore, the means for treatments, genotypes, and their interaction were compared to mark any significance by using the LSD test at the 5% level of probability.

The reduction percentage (R%) of each trait under SE, relative to the measurement under NE, was estimated per season using the equation number (2) according to [19]:

R% = 1-(Y /Y_P_) ∗100

where Y and Y_P_ are as defined above for HSI.

Pearson correlation coefficient (r) was used to study the pairwise relationship between measured traits. Two multivariate approaches were performed, including the Principle Component Analysis (PCA) and the two-way hierarchical clustering, using JMP Pro software (version 8.0; SAS Institute, Cary, NC, USA). The PCA was used to demonstrate the dimension of relationships among the measured traits. While the two-way hierarchical clustering helped to group the genotypes in association with the phenotype traits using the average linkage method. The clusters were coloured based on the K-means clustering approach.

## 3 Results and discussion

### 3.1 Meteorological data

**Figure 1:**
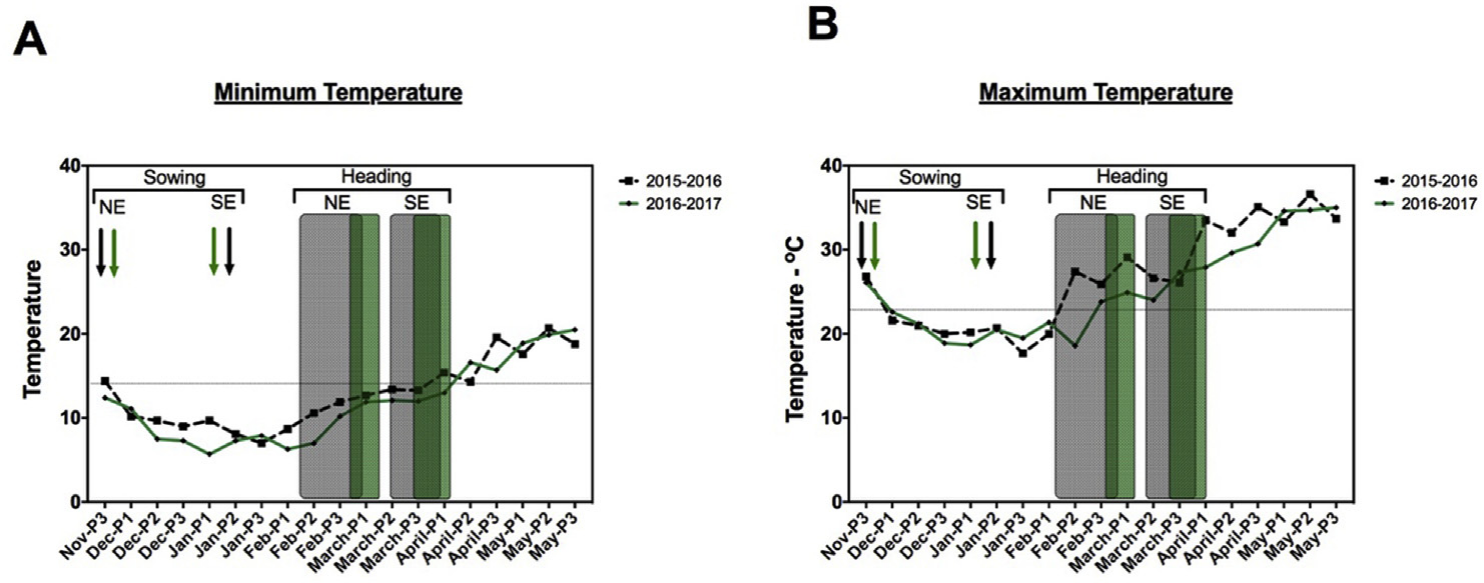
Illustrated charts to show the minimum and maximum temperatures during wheat growing successive seasons 2015/2016 (S1) and 2016/2017 (S2) according to the Egyptian Meteorological Authority - EMA. In each graph, the sowing and heading times zone of the normal environment (NE) and heat stress environment (SE) were shaded. ∗The grey lines and shaded grey zones represent during S1, while the green lines and shaded zones referred to S2. These data cover over 50 km^2^ of Giza Meteorological station.

Based on the metreological data provided, the delay of the sowing date by 53 days in S1 and 58 days in S2 has addressed the increases in temperatures more effectively than NE. Across the entire seasons under NE, the average of the minimum and maximum temperatures in S1 (11.6 °C and 25.1 °C, respectively) are higher than its values in S2 (10.1 °C and 23.3 °C, respectively). However, under SE, slight differences in values were observed between S1 (13.2 °C and 27.6 °C, respectively) and S2 (13.8 °C and 27.7 °C, respectively). That is to say, the temperature increases in S2 (3.7 °C and 4.4 °C for min. and max. temperature) were higher than S1 (1.6 °C and 2.5 °C for min. and max. temperature). The average of the minimum and maximum temperatures during the heading zone (starts from anthesis till the end of GFD) increased by 2.7 °C and 3.3 °C respectively in S1, compared to the NE, and 2.5 °C and 3.4 °C respectively in S2 (Figure 1). Through the heading-to-harvest time zones, plants experienced the highest rates of heat at both seasons.

As a result of temperature fluctuations, the difference between NE and SE in S2 was recorded as greater than that in S1, which led to the instability of some genotype performances across both seasons and helped test the mal-adaptive performance of genotypes in response to heat. However, the majority of the environmental variance (E) derived from the impact of heat was not as the seasonal variation. In agreement with the approved methodology of late sowing to expose wheat plants to temperature stress [38, 39, 40, 41], our study succeeded to distinguish the tolerant genotypes and the plastic traits to heat.

### 3.2 Analysis of variance

Due to the natural changes in stress intensity across the seasons and our evaluation of the same seed batch for two consecutive seasons, our analysis of the variance (Table 2) was performed for each season separately. Our results indicates highly significant differences (*P < 0.01*) between treatments (SE and NE) and between the genotypes for all the investigated traits. This suggests that the magnitude of differences in genotypes is sufficient to provide a scope for selecting genotypes with improved heat stress tolerance, hence such genotype can be used to create desirable genetic variability for heat stress tolerance. The environment by the genotype interaction variance (ExG) proves significant for almost all the studied traits, except DTM and GFD in S2, hence our results were in agreement with [19, 42, 43]. In addition, the coefficient of variation (CV%) values of all traits in our study shows that, GY/m^2^ and 100 KW followed by the GFD are the most variable traits in both seasons whilst the PH followed by the DTH has the least variation among the studied traits.

**Table 2:** Analysis of variance of split plot design for 20 wheat genotypes in the first (S1) and second (S2) seasons.

Season 1 (2015/2016)									
Source	DF	PH	SL	SNO	GY	KW	DTH	DTM	GFD
Rep.	2	2.44	0.5716	628.4	7302	0.1389	1.075	5.425	11.1
Environment (E)	1	8325.0∗∗	92.23∗∗	86564.4∗∗	2225192∗∗	29.67∗∗	13440.83∗∗	27331.09∗∗	2439.09∗∗
Error a	2	2.265	0.1391	21.3	2225192	0.0011	0.658	1.608	0.233
Genotype (G)	19	259.68∗∗	4.29∗∗	9849∗∗	480∗∗	0.92∗∗	90.65∗∗	89.88∗∗	16.55∗∗
E x G	19	36.35∗∗	0.80∗∗	1857.9∗∗	31510∗∗	0.23∗∗	16.90∗∗	27.75∗∗	15.04∗∗
Error b	76	1.497	0.1588	260.9	3120	0.108	3.209	3.183	5.991
C.V%		1.41	3.75	5.79	10.31	8.37	2.31	1.52	6.15
Season 2 (2016/2017)									
Source	DF	PH	SL	SNO	GYO	100 KW	DTH	DTM	GFD
Rep.	2	1.6083	0.1187	667.5	4102	0.02107	0.208	2.533	4.075
Environment (E)	1	21120.53∗∗	131.25∗∗	159286.5∗∗	2950276∗∗	15.02∗∗	25433.41∗∗	59452.01∗∗	7114.8∗∗
Error a	2	0.9083	0.0271	238.3	1305	0.06541	0.608	8.633	4.975
Genotype (G)	19	342.21∗∗	5.62∗∗	15115.3∗∗	46260∗∗	0.75∗∗	87.03∗∗	48.22∗∗	27.07∗∗
E x G	19	45.02∗∗	0.79∗∗	3961∗∗	17857∗∗	0.15∗	3.5∗	8.69 ns	6.36 ns
Error b	76	0.7496	0.3098	234.2	2470	0.0787	1.935	5.557	7.332
C.V%		0.91	4.89	3.66	8.22	6.55	1.71	1.94	6.71

PH: plant height, SL: spike length, SNO: spike number/m^2^, GY: grain yield/m^2^, KW: 100 kernel weight, DTH: days to heading, DTM: days to maturity, GFD: grain filling duration. Asterisk ∗, ∗∗ represents Significant at *P* < 0.05% and 0.01%, respectively, ns: non-significant.

### 3.3 Screening the impact of heat stress

#### 3.3.1 Phenological, morphological and agronomical parameters

Heat stress causes inconsistency in the physiological processes, biochemical reactions and cellular components, whichy can be visualized and measured in the agro-morphological and phenological traits. The heat stress impacted the plant growth and the duration of developmental stages. The heat stress during both seasons shortened DTH, DTM and GFD, compared to the NE in all studied genotypes (Tables 3 and 4). Generally, the reduction in these phenological traits in S2 (30%–32%) was higher than S1 (20%–24%) across all genotypes (Table 5), which could be due to the higher temperature differences between SE and NE that occurred in S2 (3.7 °C and 4.4 °C) than S1 (1.6 °C and 2.5 °C). This higher temperature rate in S2 also accelerated the life cycle of stressed plants by about 45 days compared to 30 days in S1, which was detected by counting DTM. In accordance with other studies, the shortening in DTH, DTM and GFD in response to heat caused a decline in yield [44, 45, 46]. This decline is mainly due to shortening the time available to build stem reserves and the time available for grain filling [6, 47]. As observed in [6], for each 1 °C increase in mean temperature, the post heading period which is shortened by 2.2 days resulted in final yield reduction. Interestingly, the genotypes that produced the highest grain yield in both seasons, such as Sids1 and Masr2, spent the longest days to maturation. Although the long duration allowed them to be exposed to a temperature peak during the season, their yield loss scored the least reduction compared to other genotypes. An explanation of this is that longer post-heading duration may allow more grain set and increase individual grain mass. Furthermore another study observed genotypes with a longer post-heading duration and more tolerance to heat stress [5]. Here, this study observed that, in both of the tolerant and susceptible genotypes, heat may negatively impact the duration of the growth stages though the destinations of this pathway are different. We observed that the susceptible genotype is more likely to face early senescence and growth limitation while the tolerant genotype is capable of limiting the loss during the vegetative stage and able to escape in order to have enough time for the grain filling duration.

**Table 3:** Means and Standard deviation of all studied phenotypic parameters under non-stress (NE) and stress (SE) environments in first season (S1).

Genotype	PH (cm)		SL (cm)		SNO (number)		GY/m^2^ (gm)		100 KW (gm)		DTH (days)		DTM (days)		GFD (days)	
	NE	SE	NE	SE	NE	SE	NE	SE	NE	SE	NE	SE	NE	SE	NE	SE
Sids 1	107.33 ± 2.08	82.50 ± 1.32	12.17 ± 0.29	11.50 ± 0.50	388.33 ± 15.18	298.33 ± 13.87	765.09 ± 39.65	560.32 ± 46.91	4.51 ± 0.29	3.52 ± 0.22	94.33 ± 0.58	75.00 ± 2.0	143.00 ± 2.0	110.00 ± 1.0	48.67 ± 1.53	35.00 ± 1.73
Sids 12	93.00 ± 1.0	77.50 ± 1.32	12.00 ± 0.50	10.50 ± 0.50	238.00 ± 8.54	191.67 ± 7.02	690.04 ± 66.44	409.52 ± 12.09	5.08 ± 0.20	3.49 ± 0.11	78.00 ± 2.0	58.00 ± 0.0	120.00 ± 0.0	95.00 ± 2.0	42.00 ± 2.0	37.00 ± 2.0
Sids 13	78.00 ± 1.32	64.17 ± 0.76	9.93 ± 0.51	8.50 ± 0.50	322.67 ± 11.24	284.00 ± 8.19	597.12 ± 27.55	355.81 ± 14.43	3.20 ± 0.16	2.86 ± 0.12	90.67 ± 3.51	67.00 ± 2.0	136.00 ± 2.0	103.00 ± 2.0	45.33 ± 1.53	36.00 ± 2.0
Masr 2	99.00 ± 1.73	97.00 ± 0.50	10.67 ± 0.76	10.50 ± 0.50	455.67 ± 20.60	310.67 ± 21.55	780.01 ± 79.19	589.33 ± 89.01	4.14 ± 0.21	3.18 ± 0.15	96.33 ± 1.15	71.00 ± 0.0	141.00 ± 1.0	106.33 ± 0.58	44.67 ± 1.53	35.33 ± 0.58
Shandwel 1	97.00 ± 2.0	80.00 ± 1.0	12.17 ± 0.29	10.00 ± 0.0	295.67 ± 16.56	270.67 ± 9.50	735.22 ± 73.71	400.08 ± 33.94	4.05 ± 0.18	3.32 ± 0.15	88.00 ± 2.0	66.33 ± 0.58	137.00 ± 2.0	100.33 ± 0.58	49.00 ± 2.0	34.00 ± 0.0
Gemmiza 9	97.50 ± 0.87	83.00 ± 1.0	12.50 ± 0.50	10.00 ± 0.50	325.00 ± 5.57	235.00 ± 25.51	715.65 ± 25.64	380.25 ± 83.31	4.26 ± 0.18	3.40 ± 0.18	94.33 ± 0.58	67.33 ± 2.08	139.00 ± 4.0	101.00 ± 2.0	44.67 ± 4.04	33.67 ± 4.04
Gemmiza 10	90.00 ± 1.0	78.50 ± 1.32	12.43 ± 0.40	10.00 ± 0.0	325.00 ± 23.26	203.33 ± 10.97	705.52 ± 72.42	320.49 ± 29.75	4.40 ± 0.44	3.29 ± 0.24	94.33 ± 0.58	69.33 ± 0.58	138.67 ± 1.15	103.00 ± 1.0	44.33 ± 1.53	33.67 ± 0.58
Gemmiza 11	95.00 ± 2.0	80.50 ± 0.50	13.00 ± 0.50	11.33 ± 0.29	261.67 ± 17.01	237.67 ± 18.01	810.77 ± 84.99	490.78 ± 59.71	5.02 ± 0.50	4.27 ± 0.38	85.33 ± 2.08	67.33 ± 1.53	130.00 ± 1.0	105.00 ± 4.0	44.67 ± 3.06	37.67 ± 3.21
Giza 171	101.00 ± 1.0	82.17 ± 0.76	11.00 ± 0.50	10.50 ± 0.50	258.00 ± 19.08	215.67 ± 9.07	645.67 ± 73.59	475.95 ± 22.05	5.30 ± 0.59	4.28 ± 0.22	84.00 ± 2.0	65.00 ± 2.0	131.00 ± 0.0	100.67 ± 0.58	47.00 ± 2.0	35.67 ± 2.52
Line 1	101.50 ± 0.87	81.67 ± 0.58	12.50 ± 0.50	10.00 ± 0.0	344.00 ± 15.0	311.00 ± 20.07	642.29 ± 61.87	380.16 ± 50.47	4.41 ± 0.38	3.48 ± 0.32	84.33 ± 1.53	66.67 ± 0.58	128.00 ± 0.0	103.33 ± 0.58	43.67 ± 2.89	36.67 ± 0.58
Line 2	99.33 ± 1.15	77.17 ± 0.76	11.17 ± 0.29	9.83 ± 0.76	319.33 ± 10.07	265.00 ± 21.66	654.16 ± 48.23	412.68 ± 56.82	4.63 ± 0.35	3.71 ± 0.40	83.67 ± 0.58	66.00 ± 0.0	127.00 ± 0.0	104.00 ± 2.0	43.33 ± 2.52	38.00 ± 2.0
Line 3	105.00 ± 1.0	85.00 ± 2.0	11.33 ± 0.29	10.00 ± 0.0	276.67 ± 14.64	240.00 ± 23.52	720.93 ± 60.76	400.35 ± 89.03	4.29 ± 0.31	3.63 ± 0.37	84.00 ± 0.0	65.00 ± 4.0	128.00 ± 1.0	102.33 ± 2.52	44.00 ± 1.0	37.33 ± 5.86
Line 4	88.50 ± 1.8	73.83 ± 0.76	10.33 ± 0.58	8.00 ± 0.50	276.67 ± 13.32	212.33 ± 8.33	570.51 ± 43.27	220.75 ± 17.66	4.63 ± 0.36	2.93 ± 0.20	90.00 ± 3.0	67.00 ± 2.0	130.33 ± 0.58	100.33 ± 0.58	40.33 ± 3.51	33.33 ± 1.53
Line 5	88.50 ± 0.50	71.17 ± 1.26	11.33 ± 0.29	8.50 ± 0.50	272.33 ± 12.66	201.00 ± 22.07	567.18 ± 50.95	340.90 ± 37.71	3.85 ± 0.35	3.45 ± 0.15	90.33 ± 0.58	66.33 ± 2.52	132.33 ± 2.31	103.33 ± 0.58	42.00 ± 2.65	37.00 ± 2.0
Line 6	84.00 ± 1.0	69.67 ± 2.08	11.20 ± 0.26	9.00 ± 0.0	278.33 ± 10.69	275.67 ± 10.79	703.11 ± 31.28	421.00 ± 20.01	4.12 ± 0.32	3.61 ± 0.39	93.33 ± 0.58	66.33 ± 0.58	133.00 ± 2.0	102.00 ± 2.0	39.67 ± 2.08	35.67 ± 2.08
Line 7	88.00 ± 2.0	71.00 ± 0.87	12.00 ± 0.00	10.33 ± 0.29	289.00 ± 26.29	274.00 ± 17.52	750.53 ± 89.60	414.47 ± 89.22	4.24 ± 0.34	2.96 ± 0.31	92.00 ± 1.0	70.33 ± 2.52	133.33 ± 2.08	99.67 ± 0.58	41.33 ± 3.06	29.33 ± 2.52
Line 8	97.00 ± 1.0	80.17 ± 1.26	11.83 ± 0.29	10.33 ± 0.29	285.00 ± 14.15	245.00 ± 23.26	740.68 ± 46.63	422.00 ± 66.25	4.49 ± 0.44	3.47 ± 0.48	85.33 ± 2.08	64.67 ± 0.58	128.00 ± 2.0	100.00 ± 3.0	42.67 ± 1.53	35.33 ± 3.51
Line 9	95.17 ± 1.26	81.00 ± 1.0	10.33 ± 0.29	8.50 ± 0.50	341.67 ± 20.01	297.00 ± 14.11	561.67 ± 72.64	391.50 ± 29.28	4.00 ± 0.28	3.05 ± 0.52	86.33 ± 0.58	65.33 ± 1.53	131.67 ± 1.53	101.33 ± 0.58	45.33 ± 1.15	36.00 ± 1.73
Line 10	96.00 ± 0.50	73.50 ± 1.32	10.83 ± 0.29	9.00 ± 0.0	286.67 ± 10.60	233.33 ± 16.26	603.21 ± 47.59	310.87 ± 34.01	5.02 ± 0.39	3.51 ± 0.45	82.00 ± 2.0	66.00 ± 3.0	128.33 ± 0.58	100.67 ± 1.15	46.33 ± 2.08	34.67 ± 2.08
Line 11	95.67 ± 0.58	73.83 ± 0.76	11.17 ± 0.29	8.50 ± 0.50	275.00 ± 16.52	239.00 ± 9.54	600.27 ± 45.82	415.46 ± 22.30	4.81 ± 0.19	3.15 ± 0.29	84.00 ± 2.0	67.33 ± 1.53	131.67 ± 2.08	102.33 ± 2.31	47.67 ± 1.53	35.00 ± 3.61
Overall Mean	94.83	78.17	11.50	9.74	305.73	252.02	677.98	405.63	4.42	3.43	88.03	66.87	132.37	102.18	44.33	35.32
LSD_0.05_ (E)	1.182		0.293		3.622		17.207		0.026		0.637		0.996		0.379	
LSD_0.05_ (G)	1.407		0.458		18.575		64.225		0.378		2.06		2.052		2.815	
LSD _0.05_ (E×G)	2.017		0.646		25.655		88.866		0.521		2.854		2.864		3.883	

NE: non-stress environment, SE: stress environment, PH: plant height, SL: spike length, SNO: spike number/m^2^, KW: kernel weight, GY: grain yield, DTH: days to heading, DTM: days to maturity, GFD: grain filling duration.

**Table 4:** Means and Standard deviation of all studied phenotypic parameters under non-stress (NE) and stress (SE) environments in second season (S2).

Genotype	PH (cm)		SL (cm)		SNO (number)		GY/m^2^ (gm)		100 KW (gm)		DTH (days)		DTM (days)		GFD (days)	
	NE	SE	NE	SE	NE	SE	NE	SE	NE	SE	NE	SE	NE	SE	NE	SE
Sids 1	124.00 ± 1.0	89.00 ± 1.0	14.50 ± 0.50	11.67 ± 0.58	440.33 ± 9.02	368.00 ± 10.0	920.83 ± 81.91	587.50 ± 80.88	4.62 ± 0.37	3.92 ± 0.30	101.00 ± 1.0	72.00 ± 2.0	150.00 ± 1.0	104.00 ± 1.0	49.00 ± 1.0	32.00 ± 3.0
Sids 12	100.00 ± 1.0	73.00 ± 1.0	13.67 ± 0.58	12.33 ± 0.58	366.00 ± 18.0	244.00 ± 19.6	856.12 ± 59.88	395.83 ± 51.58	4.92 ± 0.23	3.81 ± 0.19	89.00 ± 2.0	59.00 ± 1.0	136.33 ± 3.06	92.67 ± 0.58	47.33 ± 3.06	33.67 ± 1.53
Sids 13	92.33 ± 0.76	66.67 ± 1.15	10.50 ± 0.50	8.33 ± 0.58	517.33 ± 20.4	395.00 ± 21.93	702.08 ± 100.19	404.17 ± 77.67	3.56 ± 0.38	3.14 ± 0.36	98.00 ± 1.0	68.00 ± 2.0	143.33 ± 1.53	100.33 ± 0.58	45.33 ± 2.08	32.33 ± 1.53
Masr 2	117.33 ± 0.58	102.83 ± 0.76	11.33 ± 0.58	10.50 ± 0.50	509.33 ± 21.13	427.00 ± 21.63	802.04 ± 77.62	610.88 ± 45.62	4.22 ± 0.40	3.87 ± 0.27	100.00 ± 1.0	73.00 ± 1.0	148.67 ± 3.21	102.67 ± 2.52	48.67 ± 3.51	29.67 ± 3.21
Shandwel 1	110.33 ± 0.29	76.67 ± 0.58	13.50 ± 0.50	11.67 ± 0.29	437.00 ± 28.16	264.33 ± 17.24	806.25 ± 79.17	379.50 ± 53.86	4.21 ± 0.30	3.82 ± 0.17	96.00 ± 2.0	66.00 ± 2.0	145.00 ± 2.0	102.33 ± 0.58	49.00 ± 0.0	36.33 ± 2.52
Gemmiza 9	109.83 ± 0.76	87.00 ± 1.0	13.33 ± 0.58	9.67 ± 0.58	469.33 ± 19.50	359.33 ± 8.62	895.91 ± 77.62	420.22 ± 29.50	5.20 ± 0.31	3.87 ± 0.11	100.00 ± 1.0	71.00 ± 2.0	146.00 ± 4.0	101.00 ± 2.0	46.00 ± 3.61	30.00 ± 2.0
Gemmiza 10	108.83 ± 0.76	91.33 ± 0.58	12.00 ± 0.0	9.67 ± 0.58	412.33 ± 15.50	400.00 ± 16.09	716.67 ± 39.61	454.17 ± 27.74	4.45 ± 0.42	3.99 ± 0.12	96.00 ± 2.0	69.00 ± 1.0	145.00 ± 0.0	104.00 ± 1.0	49.00 ± 2.0	35.00 ± 0.0
Gemmiza 11	109.67 ± 0.58	80.00 ± 1.0	13.67 ± 0.58	11.50 ± 0.50	410.00 ± 8.72	361.33 ± 12.50	843.88 ± 33.79	441.67 ± 47.02	5.11 ± 0.29	4.41 ± 0.10	93.00 ± 1.0	61.00 ± 0.0	144.33 ± 4.51	94.00 ± 2.0	51.33 ± 5.13	33.00 ± 2.0
Giza 171	112.00 ± 1.0	87.00 ± 1.0	13.17 ± 0.67	11.33 ± 0.29	360.00 ± 9.0	353.33 ± 13.32	585.42 ± 21.79	514.58 ± 15.28	5.26 ± 0.38	4.75 ± 0.15	93.33 ± 0.58	65.33 ± 0.58	143.33 ± 2.52	99.33 ± 2.08	50.00 ± 2.0	34.00 ± 2.65
Line 1	111.67 ± 1.15	83.67 ± 0.58	12.67 ± 0.58	10.83 ± 0.29	443.33 ± 6.66	395.33 ± 5.51	618.75 ± 13.04	327.08 ± 12.69	4.53 ± 0.33	3.84 ± 0.19	92.00 ± 2.0	61.00 ± 2.0	140.00 ± 2.0	94.00 ± 2.0	48.00 ± 0.0	33.00 ± 0.0
Line 2	113.67 ± 0.58	77.33 ± 0.58	12.17 ± 0.29	9.33 ± 0.29	409.00 ± 12.49	347.00 ± 10.44	650.00 ± 37.83	338.75 ± 18.97	4.86 ± 0.32	3.94 ± 0.30	91.00 ± 2.0	62.00 ± 1.0	143.00 ± 3.0	96.00 ± 4.0	52.00 ± 5.0	34.00 ± 4.58
Line 3	117.50 ± 0.50	88.00 ± 0.0	13.83 ± 0.76	10.33 ± 0.29	544.00 ± 9.17	408.00 ± 18.36	733.00 ± 28.61	401.38 ± 17.58	4.83 ± 0.21	4.00 ± 0.33	91.33 ± 1.53	61.00 ± 1.0	144.00 ± 2.0	95.00 ± 1.0	52.67 ± 2.89	34.00 ± 0.0
Line 4	103.00 ± 1.0	79.67 ± 0.58	11.33 ± 0.58	9.67 ± 0.58	491.00 ± 18.52	419.00 ± 13.53	696.58 ± 54.51	325.75 ± 18.42	4.50 ± 0.29	3.49 ± 0.32	100.00 ± 0.0	73.00 ± 0.0	143.33 ± 2.52	101.33 ± 3.21	43.33 ± 2.52	28.33 ± 3.21
Line 5	103.67 ± 0.58	72.67 ± 0.58	12.00 ± 0.0	10.00 ± 1.0	417.33 ± 12.66	399.00 ± 16.64	589.58 ± 19.37	391.67 ± 23.04	4.00 ± 0.32	3.71 ± 0.29	98.00 ± 1.0	67.33 ± 0.58	143.00 ± 1.0	98.00 ± 2.0	45.00 ± 1.73	30.67 ± 2.52
Line 6	95.33 ± 0.58	72.33 ± 0.58	12.00 ± 1.0	9.17 ± 0.29	465.33 ± 13.50	456.00 ± 12.12	920.83 ± 62.71	531.25 ± 38.24	4.75 ± 0.23	4.03 ± 0.24	100.00 ± 2.0	69.00 ± 2.0	144.00 ± 2.0	100.00 ± 1.0	44.00 ± 0.0	31.00 ± 1.73
Line 7	100.33 ± 0.58	75.00 ± 1.0	12.17 ± 0.76	10.83 ± 0.29	521.00 ± 19.0	399.00 ± 15.52	825.00 ± 81.64	422.92 ± 49.75	4.49 ± 0.32	3.88 ± 0.36	98.00 ± 1.0	69.00 ± 0.0	142.00 ± 1.0	101.00 ± 2.65	44.00 ± 1.0	32.00 ± 2.65
Line 8	108.67 ± 1.53	84.67 ± 1.53	12.33 ± 0.58	10.50 ± 0.50	550.33 ± 4.51	471.00 ± 10.54	945.83 ± 23.46	572.07 ± 27.73	4.87 ± 0.14	4.00 ± 0.22	95.33 ± 0.58	66.00 ± 2.0	144.00 ± 2.0	100.00 ± 5.0	48.67 ± 1.53	34.00 ± 7.0
Line 9	110.33 ± 2.08	88.67 ± 1.15	11.50 ± 0.50	9.67 ± 0.58	447.00 ± 27.0	438.00 ± 5.29	652.08 ± 58.43	504.17 ± 12.25	4.21 ± 0.17	4.06 ± 0.23	95.00 ± 2.0	66.33 ± 0.58	145.00 ± 2.65	100.33 ± 0.58	50.00 ± 1.0	34.00 ± 1.0
Line 10	105.67 ± 0.58	78.00 ± 0.0	11.67 ± 0.29	10.17 ± 0.29	443.00 ± 13.53	308.00 ± 15.87	701.67 ± 50.36	350.25 ± 43.54	5.14 ± 0.35	4.23 ± 0.22	92.33 ± 0.58	65.00 ± 1.0	142.00 ± 0.0	99.00 ± 3.0	49.67 ± 0.58	34.00 ± 2.65
Line 11	106.33 ± 0.58	76.33 ± 0.58	11.33 ± 0.58	9.67 ± 0.76	447.33 ± 8.33	430.33 ± 22.81	762.50 ± 27.46	579.29 ± 32.35	4.95 ± 0.05	3.77 ± 0.24	95.00 ± 0.0	68.00 ± 1.0	143.00 ± 3.0	100.00 ± 2.0	48.00 ± 3.0	32.00 ± 2.65
Overall Mean	108.03	81.49	12.43	10.34	455.02	382.15	761.25	447.66	4.63	3.93	95.72	66.60	143.77	99.25	48.05	32.65
LSD_0.05_ (E)	0.749		0.129		12.127		28.381		0.201		0.613		2.308		1.752	
LSD_0.05_ (G)	0.996		0.64		17.597		57.144		0.323		1.599		2.711		3.114	
LSD_0.05_ (EXG)	1.416		0.884		24.897		79.822		0.454		2.222		ns		ns	

NE: non-stress environment, SE: stress environment, PH: plant height, SL: spike length, SNO: spike number**/**m^2^, KW: kernel weight, GY: grain yield, DTH: days to heading, DTM: days to maturity, GFD: grain filling duration, ns: non-significant.

**Table 5:** The reduction percentage (R%) for each studied phenotypic parameter in both seasons (S1 and S2).

Genotypes	PH		SL		SNO		GY/m^2^		100 KW		DTH		DTM		GFD	
	S1	S2	S1	S2	S1	S2	S1	S2	S1	S2	S1	S2	S1	S2	S1	S2
Sids 1	23.14	28.23	5.48	19.54	23.18	16.43	26.76	36.20	21.95	15.15	20.49	28.71	23.08	30.67	28.08	34.69
Sids 12	16.67	27.00	12.50	9.76	19.47	33.33	40.65	53.76	31.30	22.56	25.64	33.71	20.83	32.03	11.90	28.87
Sids 13	17.74	27.80	14.43	20.63	11.98	23.65	40.41	42.43	10.63	11.80	26.10	30.61	24.26	30.00	20.59	28.68
Masr 2	2.02	12.36	1.56	7.35	31.82	16.16	24.45	23.83	23.19	8.29	26.30	27.00	24.59	30.94	20.90	39.04
Shandwel 1	17.53	30.51	17.81	13.58	8.46	39.51	45.58	52.93	18.02	9.26	24.62	31.25	26.76	29.43	30.61	25.85
Gemmiza 9	14.87	20.79	20.00	27.50	27.69	23.44	46.87	53.10	20.19	25.58	28.62	29.00	27.34	30.82	24.63	34.78
Gemmiza 10	12.78	16.08	19.57	19.44	37.44	2.99	54.57	36.63	25.23	10.34	26.50	28.13	25.72	28.28	24.06	28.57
Gemmiza 11	15.26	27.05	12.82	15.85	9.17	11.87	39.47	47.66	14.94	13.70	21.09	34.41	19.23	34.87	15.67	35.71
Giza 171	18.65	22.32	4.55	13.92	16.41	1.85	26.29	12.10	19.25	9.70	22.62	30.00	23.16	30.70	24.11	32.00
Line 1	19.54	25.07	20.00	14.47	9.59	10.83	40.81	47.14	21.09	15.23	20.95	33.70	19.27	32.86	16.03	31.25
Line 2	22.32	31.96	11.94	23.29	17.01	15.16	36.91	47.88	19.87	18.93	21.12	31.87	18.11	32.87	12.31	34.62
Line 3	19.05	25.11	11.76	25.30	13.25	25.00	44.47	45.24	15.38	17.18	22.62	33.21	20.05	34.03	15.15	35.44
Line 4	16.57	22.65	22.58	14.71	23.25	14.66	61.31	53.24	36.72	22.44	25.56	27.00	23.02	29.30	17.36	34.62
Line 5	19.59	29.90	25.00	16.67	26.19	4.39	39.90	33.57	10.39	7.25	26.57	31.29	21.91	31.47	11.90	31.85
Line 6	17.06	24.13	19.64	23.61	0.96	2.01	40.12	42.31	12.38	15.16	28.93	31.00	23.31	30.56	10.08	29.55
Line 7	19.32	25.25	13.89	10.96	5.19	23.42	44.78	48.74	30.19	13.59	23.55	29.59	25.25	28.87	29.03	27.27
Line 8	17.35	22.09	12.68	14.86	14.04	14.42	43.03	39.52	22.72	17.86	24.22	30.77	21.88	30.56	17.19	30.14
Line 9	14.89	19.64	17.74	15.94	13.07	2.01	30.30	22.68	23.75	3.56	24.32	30.18	23.04	30.80	20.59	32.00
Line 10	23.44	26.18	16.92	12.86	18.60	30.47	48.46	50.08	30.08	17.70	19.51	29.60	21.56	30.28	25.18	31.54
Line 11	22.82	28.21	23.88	14.71	13.09	3.80	30.79	24.03	34.51	23.84	19.84	28.42	22.28	30.07	26.57	33.33
Mean	17.57	24.56	15.25	16.82	17.57	16.01	40.17	41.19	22.49	15.27	24.04	30.42	22.80	30.96	20.34	32.05

S1 = Season 1, S2 = Season 2, PH: plant height, SL: spike length, SNO: spike number**/**m^2^, KW: kernel weight, GY: grain yield, DTH: days to heading, DTM: days to maturity, GFD: grain filling duration.

Across all studied genotypes, PH mean was reduced due to heat stress with an average of 17.57% reduction in S1, and 24.56% in S2 (Table 5). Previous studies by [48] reported a 11.9% reduction in plant height which is caused by 2 °C warming above the ambient crop canopy temperature that is applied by a free-air controlled enhancement system. For instance [49], recorded a reduction in PH by 34% and 15% when wheat was planted on 1^st^ January and 30^th^ November instead of 16^th^ November. Furthermore [50], found a mean reduction of 13.42% from a delay of 30 days. That impact may due to the influence of the vegetative stage that stops vegetative development and shortens the size of the organ developed [51]. However, the relationship between plant height and heat tolerance is still unclear. Phenotypically under NE, Sids1 was recorded as the tallest genotype while Sids13 was the shortest (Tables 3 and 4). Whilst both genotypes scored significant reductions in PH under SE conditions, Sids13 (the shortest genotype) scored greater reduction in grain yield than Sids1. Also, Masr2 was amongst the tallest genotypes. It scored the least reductions in plant height of 2.02 and 12.36% in S1 and S2, respectively (Table 5) and the best grain yield and HSI. In the same context, but under drought stress, high plasticity was noticed in tall plants [52], which make a highlight of a possible correlation between plant height and heat tolerance which needs further investigation.

When plants experienced early leaf senescence during vegetative stage in response to heat stress, the spikes had a substitutive role in rescuing photosynthesis inhibition. Thus, the spike length is an important trait [53] because less reduction may help keep enough numbers of spikelet and limit grain yield reduction [54]. In this study, the SL reduction was observed on all genotypes with an overall reduction of 15.25% and 16.82% in S1 and S2, respectively. While the reduction in SL was recorded in all genotypes, the lowest reduction was seen in Masr2 in both seasons (1.56 and 7.35% for S1 and S2, respectively). Among different stress types, the spike length generally shows a significant variation, which confirmed the chance that the spike length is a polygenic character [55, 56].

As an indication for detecting productive tillers, the spike number/m^2^ (SNO) was counted. We observed that the overall reduction reached 17.57% and 16.01% during S1 and S2, respectively. The temperature during the day (about 30 °C) and night (about 25 °C) may have severe effects on the leaf development and productive tiller formation in wheat [57]. The SNO performance varied across seasons for each genotype. However, the lowest reduction in SNO was found across the genotypes in Line6 which recorded 0.96 and 2.01% in S1 and S2, respectively. That genotype was not performing well under stress. The variation in genotype performance due to heat stress intensity is one of the main difficulties in breeding for heat tolerance [58]. We found that, in response to heat stress, the susceptible plant was not able to limit the duration of DTH that ended with long vegetation stage and short GFD and finally great reduction in grain yield.

**Figure 2:**
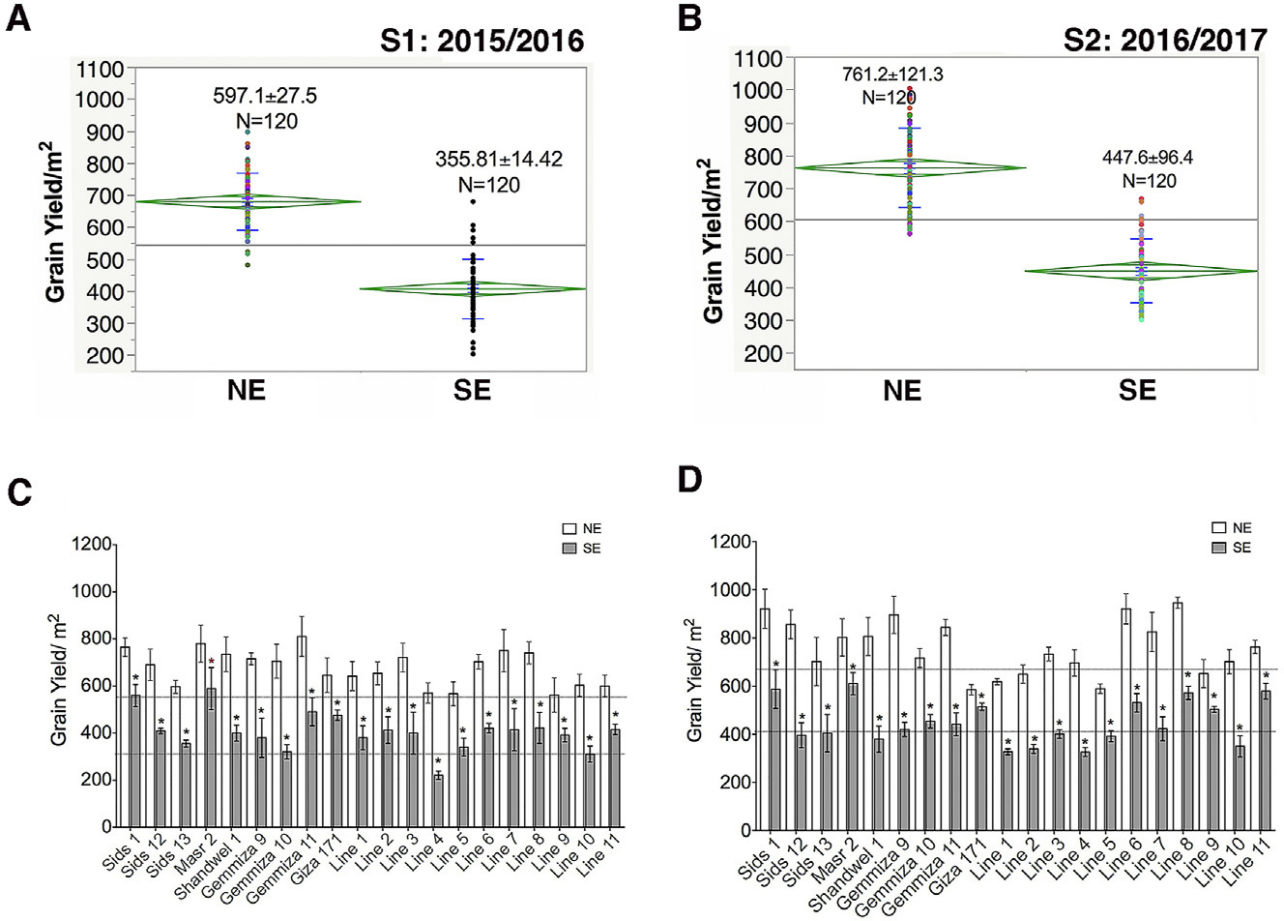
Impact of heat stress on the grain yield/m^2^. Means ± Standard deviation (SD) among all genotypes in S1 (A), and S2 (B), and the overall mean of each genotype in S1 (C) and S2 (D). Asterisk ∗ referred in each genotype to the significance in the yield under SE relative to the NE.

Since grain yield is the end product of the plant and the final destination to visualize the consequences of heat impact, thus we evaluated two traits that are related to grain weight (GY/m^2^ and 100 KW). We found that there was an overall loss in grain yield (GY/m^2^) around 40.17% in S1 (Figure 2-A) and 41.19% in S2 (Figure 2-B) and (Table 5). In agreement with others who found that delaying sowing date by 60 days may cause reduction in grain yield to reach 28.3–60.3% [20], we recorded a reduction that ranged from 24.45 to 61.31% and from 12.10 to 53.76 %, in S1 and S2, respectively. The total loss in grain yield/m^2^ due to late sowing conditions was documented intensively in the literature [6, 40, 42, 43, 59] and detected in both adaptive and non-adaptive genotypes. The issue identified here is to distinguish the significant difference in the rate of reduction and detect the stability across seasons which can point to the existence of an adaptive mechanism towards heat tolerance. In this study, the loss varied across the genotypes (Figure 2-C and D). In S1,Masr2, Giza171 and Sids1 scored the least yield loss (24.45%, 26.29% and 26.76%, respectively) while the highest reduction (61.31%) was in Line4. In S2, Giza171 and Masr2 still recorded the least reduction (12.1% and 23.83%, respectively), in addition to Line9 (22.68%). Masr2 and Sids1 scored stability in yield performance under SE in both seasons.

By comparing these findings with 100 KW trait, we can still see an overall reduction in the consecutive seasons which reached 22.49%, and 15.27%, respectively (Table 5). A study states that grain loss in wheat reached 13.3% and 5.56% by increasing the temperature from 28 °C to 44 °C and from 31.5 °C to 43.3 °C respectively [60]. In the meantime, the performance of genotypes with GY/m^2^ was not the same with 100 KW. In both seasons, it was observed that genotypes such as Masr2 and Sids1, in which the values for the 100 KW were moderate, performed well by producing the highest grain yields. These results are in accordance with [61].

### 3.4 Pollen grain viability

**Figure 3:**
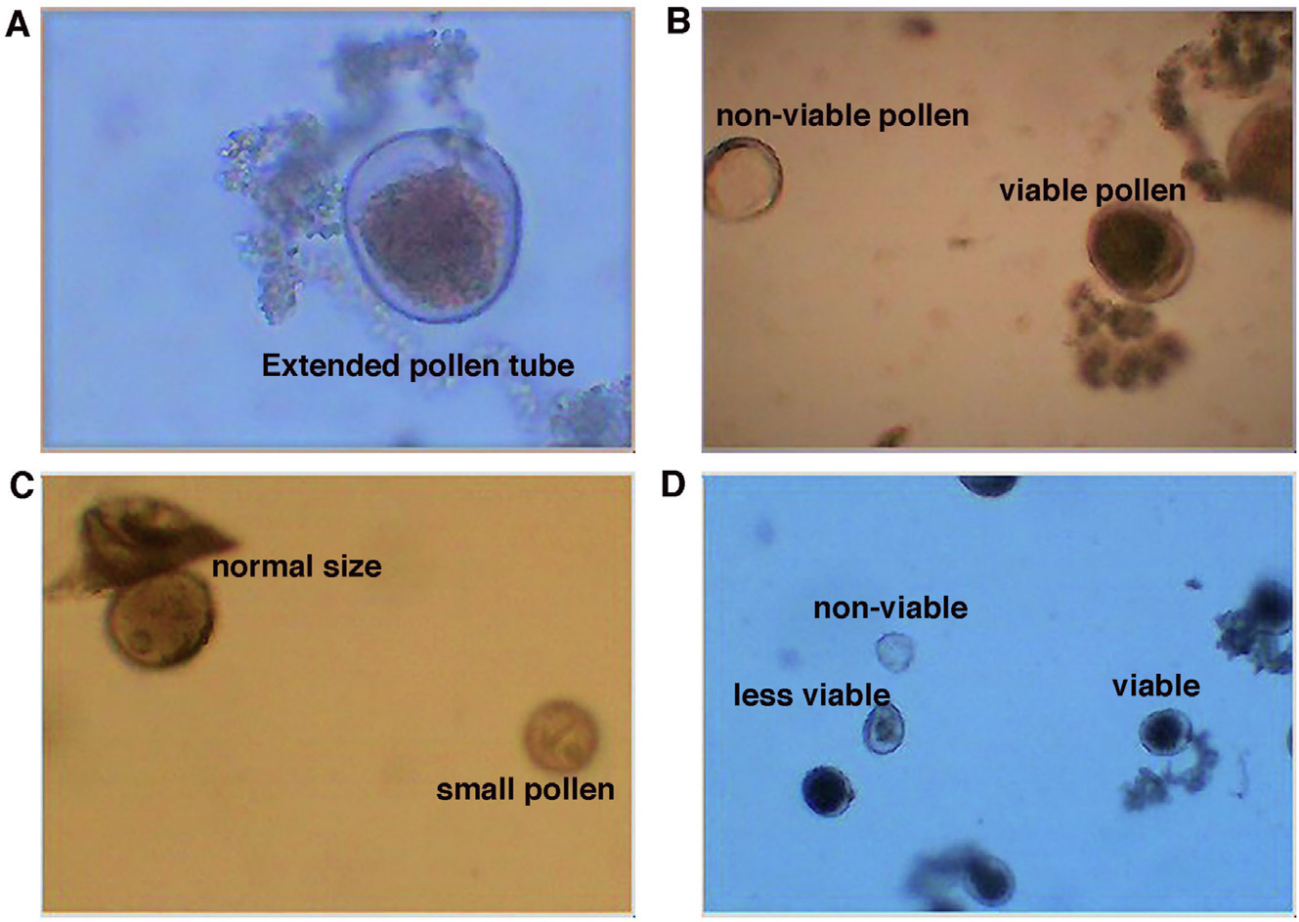
Examples of the examined pollen grains. (A) viable pollen grain extends its pollen tube, (B) viable pollen grain against non-viable pollen, (C) showing viable pollen in different size (the normal size of pollen versus smaller size that recorded in Masr 2 variety), and (D) three forms detected of pollen viability; viable, less viable.

**Figure 4:**
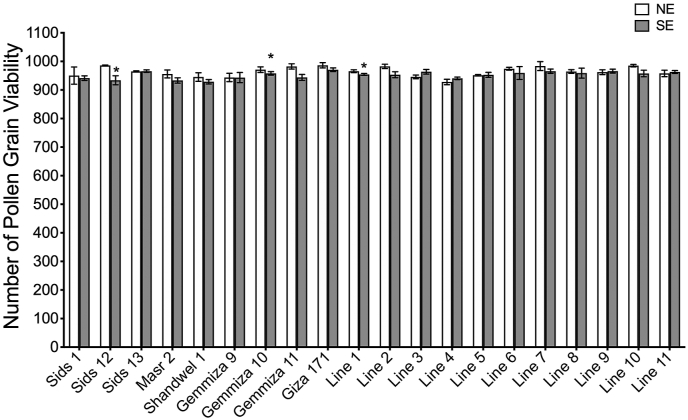
Means ± Standard Deviation (SD) of viable pollen grains for each genotype under both NE and SE conditions, Asterisk ∗ represents Significant at P = 0.05%.

At head emergence, pollens were examined to phenotype its viability, which appeared in extended pollen tubes or dark-coloured pollens (Figure 3-A, B, and C). Generally, the results reveal the limited impact of heat on pollen grain viability with slight differences among the genotypes. The heat stress caused a reduction in the number of the studied genotypes, namely Sids1, Sids12, Masr2, Shandwel1, Gemmiza10, Gemmiza11, Giza171, Line1, Line2, Line6, Line7, Line8, and Line10. However, the reduction was only significant in Sids12, Gemmiza10, and Line1 (Figure 4). The reduction in pollen viability in wheat plants as a result of heat stress has been reported previously [27, 62, 63]. The reduction in pollen grain viability after temperature elevation was indicated and referred to deformation in tapetal cells. However, these cells are responsible for nutrient translocation to the developing pollen, and lead to pollen viability or deformation [63].

Data also reveals that some genotypes do not show any change in the viable pollen numbers in response to heat stress. Examples of these genotypes are Sids13, Gemmiza9, line5, and Line9. Unexpectedly, some other genotypes (namely line3, Line4, and Line11) showed an increase in viable pollen. In response to heat conditions, the viable pollen grains of Masr2 were observed in smaller sizes than other genotypes (Figure 3-C). The genotypes that maintain their pollen viability are likely to succeed in supplying the pollen grains with an adequate amount of nutrient, depending on stored starch in plants as an energy source in a self-defense system to encourage normal development [64]. Moreover, genotypes that showed enhancement in the pollen viability in response to the temperature elevation may be explained by the interference of some genetic factors that may improve the behaviour of the stressed plant to keep its life under such stress. For example, starch biosynthesis during the pollen maturation process is considered a quality controller for pollen because starch provides pollen with the necessary energy for germination and serves as a checkpoint of pollen maturity. When looking at the grain yield reduction in S2, we found that the three genotypes that showed a significant reduction in pollen grain viability, i.e. Sids12, Gemmiza10, and Line1, gave 53.76%, 36.63%, and 47.14%, respectively. Lower floret fertility leads to a reduction in grain number, which can cause a significant loss in grain yield [27, 63].

### 3.5 Germinability

**Figure 5:**
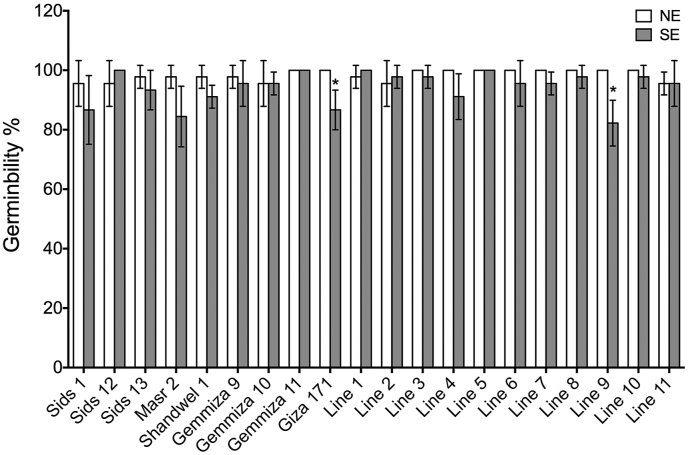
Means ± Standard Deviation (SD) of the germination rate or the germinability of seeds obtained from both normal (NE) and stress (SE) environments. Asterisk ∗ represents the significance at P = 0.05.

After harvesting, seeds that were previously exposed to heat stress during S2 were tested *in vitro* to assess the impact of heat on their germination ability, compared to the non-stressed seeds. Among all genotypes, germinability was reduced from 98.33% to 94.22% in seeds obtained from SE, compared to those resulted under NE. An insignificant reduction in germinability was recorded in 13 genotypes, while the reduction in Giza171 and Line9 was significant (Figure 5). Increasing temperature following fertilization negatively affects grain development [65, 66, 67]. On the other hand, heatwaves caused an increase in germinability in each of Sids12, Line1, and Line2 when compared to the seeds obtained from NE. Another study observed an enhancement of the germinability in response to moderate heat stress (35 °C) compared to the controlled or severe stress (39 °C), which may be due to the accumulation of starch and hormone homeostasis through seed priming [68]. Another explanation is that the germinability procedure may act as a seed priming that induces the solubility and mobility of integral cellular compounds [69, 70]. In the remaining four genotypes (Gemmiza10, Gemmiza11, Line5, and Line11), our results showed that the germinability was not affected. These findings were also observed in previous wheat studies, which found no effects on germination ability by heat stress treatment either in the seeds of the main spike or the side spike [71]. The limited impact on the germinability and pollen grain viability may be attributed to the nature of our field trials that shows less severe response to heat stress, compared to pot experiments. [72] explained that the variation that may be seen between pots and field experiments may be due to the higher temperature which the roots in pots experiments experience than field experiments.

Due to the slight differences between studied genotypes and treatments in both pollen grain viability and germinability, making them not significant to considered as reliable traits to remark the tolerance genotypes, both traits were not part of account in genotype evaluation.

## 4 Heat susceptibility index (HSI)

HSI was calculated for each of the following traits: PH, SL, SNO, 100 KW, and GY/m^2^ as shown in Table 6. The Masr 2 genotype had the lowest HSI value for the plant height and spike length in all genotypes and seasons, indicating high plasticity to high temperature. With respect to SNO trait, Line6 genotype had the least scored values (0.05) in S1 while Giza171 (0.12), Line6 and Line9 (0.13) displayed the least values in S2. Most of the genotypes scored HSI values around and above 1.00 for the 100 KW, with the exception of Shandwel1, Gemmiza11, Giza171 and Line6 which scored a moderate value in both seasons. Only Line 5 recorded a low HSI value in both seasons. Significant changes in HSI values of 100 KW were observed in Masr2, Gemmiza10 and Line9 between S1 and S2.

**Table 6:** Calculated values of Heat susceptibility index (HSI) for grain yield and the other parameters for all the studied wheat genotypes during S1 and S2.

Genotypes	PH		SL		SNO		100 KW		GY/m^2^	
	S1	S2	S1	S2	S1	S2	S1	S2	S1	S2
Sids 1	1.32	1.15	0.36	1.16	1.32	1.03	0.98	0.99	0.67	0.88
Sids 12	0.95	1.10	0.82	0.58	1.11	2.08	1.39	1.48	1.01	1.31
Sids 13	1.01	1.13	0.95	1.23	0.68	1.48	0.47	0.77	1.01	1.03
Masr 2	0.11	0.50	0.10	0.44	1.81	1.01	1.03	0.54	0.61	0.58
Shandwel 1	1.00	1.24	1.17	0.81	0.48	2.47	0.80	0.61	1.13	1.28
Gemmiza 9	0.85	0.85	1.31	1.63	1.58	1.46	0.90	1.68	1.17	1.29
Gemmiza 10	0.73	0.65	1.28	1.16	2.13	0.19	1.12	0.68	1.36	0.89
Gemmiza 11	0.87	1.10	0.84	0.94	0.52	0.74	0.66	0.90	0.98	1.16
Giza 171	1.06	0.91	0.30	0.83	0.93	0.12	0.86	0.64	0.65	0.29
Line 1	1.11	1.02	1.31	0.86	0.55	0.68	0.94	1.00	1.02	1.14
Line 2	1.27	1.30	0.78	1.38	0.97	0.95	0.88	1.24	0.92	1.16
Line 3	1.08	1.02	0.77	1.50	0.75	1.56	0.68	1.13	1.11	1.10
Line 4	0.94	0.92	1.48	0.87	1.32	0.92	1.63	1.47	1.53	1.29
Line 5	1.11	1.22	1.64	0.99	1.49	0.27	0.46	0.47	0.99	0.81
Line 6	0.97	0.98	1.29	1.40	0.05	0.13	0.55	0.99	1.00	1.03
Line 7	1.10	1.03	0.91	0.65	0.30	1.46	1.34	0.89	1.11	1.18
Line 8	0.99	0.90	0.83	0.88	0.80	0.90	1.01	1.17	1.07	0.96
Line 9	0.85	0.80	1.16	0.95	0.74	0.13	1.06	0.23	0.75	0.55
Line 10	1.33	1.07	1.11	0.76	1.06	1.90	1.34	1.16	1.21	1.22
Line 11	1.30	1.15	1.57	0.87	0.75	0.24	1.53	1.56	0.77	0.58

S1 = Season 1, S2 = Season 2, PH: plant height, SL: spike length, SNO: spike number/m^2^, KW: kernel weight, GY: grain yield.

The HSI corresponding to the trait of GY/m^2^, 10 genotypes - namely Sids12, Sids13, Shandwel1, Gemmiza9, Line1, Line3, Line4, Line6, Line7, and Line10 - had values above 1.0 in both seasons. Particularly, Line4 showed the highest HSI values in S1 and one of the highest values in S2 (Table 6). In S1, four genotypes, Masr2, Giza171, Sids1 and Line 9, showed the lowest HSI values (0.61, 0.65, 0.67 and 0.75, respectively). In S2, three of the previous four, i.e.Giza171, Line9 and Masr2, still had the lowest values (0.29, 0.55 and 0.58, respectively).

The categorisation of wheat genotypes into highly tolerant, tolerant and susceptible to high temperature stress using the HSI scale under field conditions had already been investigated [39, 43, 50, 59, 73]. These studies concluded that the smaller HIS values (<1.00) indicate better thermal tolerance [37]. In our study, the genotypes which showed the lowest HSI values of GY/m^2^, such as Masr2, Giza171, Sids1 and line9, also indicated the highest GY/m^2^ and had the best performance under heat stress conditions. In addition, this index may describe the stability in the yield under heat stress [60].

## 5 Pairwise correlation

The correlation coefficient was performed to investigate the pairwise relationships between the eight studied phenological traits in S1 and S2 (Tables 7 and 8). It particularly emphasised that grain yield is the most important trait for breeders. Each table was split diagonally to observe the two environmental conditions in the same season. The GY/m^2^ correlated significantly with DTH in both seasons (r = 0.28, *P* = 0.033 in S1; r = 0.38, *P* = 0.03 in S2) under SE. Whilst a very significant correlation was observed between GY/m^2^ and DTM in S1 (r = 0.43, *P* = 0.0006), this was lost in S2 under SE. The association between the different traits that represented the grain yield, as well as DTH, DTM and GFD varies in the literature. Previous studies found that DTH has a weak or negative correlation with GY/m^2^ under warm conditions [5, 6, 42, 74], indicating that the plastic performance is sensitive to long DTH or late maturation. Others confirmed the correlation between grain yield and DTH and DTM, and that the adapted genotypes may have long pre-heading and GFD [75, 76]. In this case, the crop may be still economically able to produce high GY/m^2^ regardless of the endurable heat stress. This suggests that the days to maturity could be favourably selected for an enhanced grain yield, except under intense and protracted heat stress especially during the late growing season. Under intense heat stress, fast-maturing genotypes will be ideal when employing escape mechanisms to avoid the prolonged terminal heat stress that normally occurs, particularly in the tropics and sub-tropics [76]. This indicates that early maturing cultivars are preferable to escape heat stress injury that occurs at the end of the growing season. With respect to GFD, a non-significant correlation with GY/m^2^ was shown under SE in both seasons. Under late sowing, a non-significant correlation between GY and GFD in S1 changed to significant negative correlation in S2 [42].

**Table 7:** The correlation coefficient for the studied traits in season 2015/2016 (S1) under each of the normal and stress environments.

Season	PH	SL	SNO	GY/m^2^	100 KW	DTH	DTM	GFD
PH	**1.00**	**0.32∗**	**0.26**	**0.26**	**0.39∗∗**	**-0.24**	**0.01**	**0.40∗∗**
SL	0.53∗∗	**1.00**	**-0.08**	**0.54∗∗**	**0.24**	**0.03**	**0.02**	**-0.02**
SNO	0.24	0.14	**1.00**	**0.32∗**	**-0.29∗**	**0.56∗∗**	**0.63∗∗**	**0.20**
GY/m^2^	0.54∗∗	0.64∗∗	0.55∗∗	**1.00**	**0.18**	**0.25**	**0.24**	**0.02**
100 KW	0.22	0.39∗∗	-0.25	0.21	**1.00**	**-0.48∗∗**	**-0.39∗∗**	**0.09**
DTH	0.16	0.19	0.42∗∗	0.28∗	-0.1	**1.00**	**0.83∗∗**	**-0.16**
DTM	0.25∗	0.25	0.45∗∗	0.43∗∗	0.07	0.64∗∗	**1.00**	**0.42∗∗**
GFD	0.09	0.05	0.00	0.16	0.20	-0.48∗∗	0.35∗∗	**1.00**

Note: Bold numbers refer to r values under normal environment (NE), while normal values under the stress environment (SE). Asterisk ∗, ∗∗ represents Significant at P <0.05% and 0.01%, respectively.

**Table 8:** The correlation coefficient for the studied traits in season 2016/2017 (S2) under each of the normal and stress environments.

Season	PH	SL	SNO	GY/m^2^	100 KW	DTH	DTM	GFD
PH	**1.00**	**0.48∗∗**	**-0.07**	**-0.005**	**0.23**	**-0.07**	**0.46∗∗**	**0.54∗∗**
SL	0.17∗∗	**1.00**	**-0.26∗**	**0.33∗**	**0.37∗∗**	**-0.19**	**0.09**	**0.29∗**
SNO	0.24	-0.49∗	**1.00**	**0.37∗∗**	**-0.25**	**0.37∗∗**	**0.21**	**-0.19**
GY/m^2^	0.43	0.05∗	0.49∗∗	**1.00**	**0.22**	**0.28∗**	**0.17**	**-0.13**
100 KW	0.31	0.37∗∗	-0.11	0.16	**1.00**	**-0.29∗**	**-0.10**	**0.22**
DTH	0.26	-0.31	0.41∗∗	0.38∗	-0.25∗	**1.00**	**0.55∗∗**	**-0.53∗∗**
DTM	0.27∗∗	-0.22	0.23	0.36	-0.11	0.72∗∗	**1.00**	**0.41∗∗**
GFD	-0.03∗∗	0.16	0.29	-0.08	0.21	-0.51∗∗	0.23∗∗	**1.00**

Note: Bold numbers refer to r values under normal environment (NE), while normal values under the stress environment (SE). Asterisk ∗, ∗∗ represents Significant at P <0.05% and 0.01%, respectively.

Furthermore, GY/m^2^ correlated significantly with PH in S1 (r = 0.54; *P < 0.0001*) but it turned to insignificant in S2 (r = 0.43 *P = 0.97*) under SE. Some previous studies emphasised that tall plants are associated with the plastic performance under drought stress [52], and lodging during high rainfall seasons [77]. The usage of PH trait for genotype selection is still debatable [52]. In our study, the correlation between GY/m^2^ and SL under SE was significant in both seasons (r = 0.64, P < 0.0001 in S1; r = 0.05, P = 0.011 in S2), which indicates its strong role in the evaluation of the genotypes. Despite the differences in temperature between the two growing seasons, the SNO showed a significant correlation with the GY/m^2^ under both NE and SE, which is consistent with others [78]. The 100KW trait has an insignificant correlation with GY/m^2^ and a negative correlation with SNO, which faded the role of the trait in genotype selection. This is in accordance with [48]who found that this trait did not vary statistically between stressed and non-stressed plants to heat and did not show correlation with GY. Generally, this study noted that all correlational relationships with grain yield was found to be weak or moderate, and confirmed that GY was the most effective criteria to evaluate the heat stress tolerance.

## 6 Multivariate analyses

**Figure 6:**
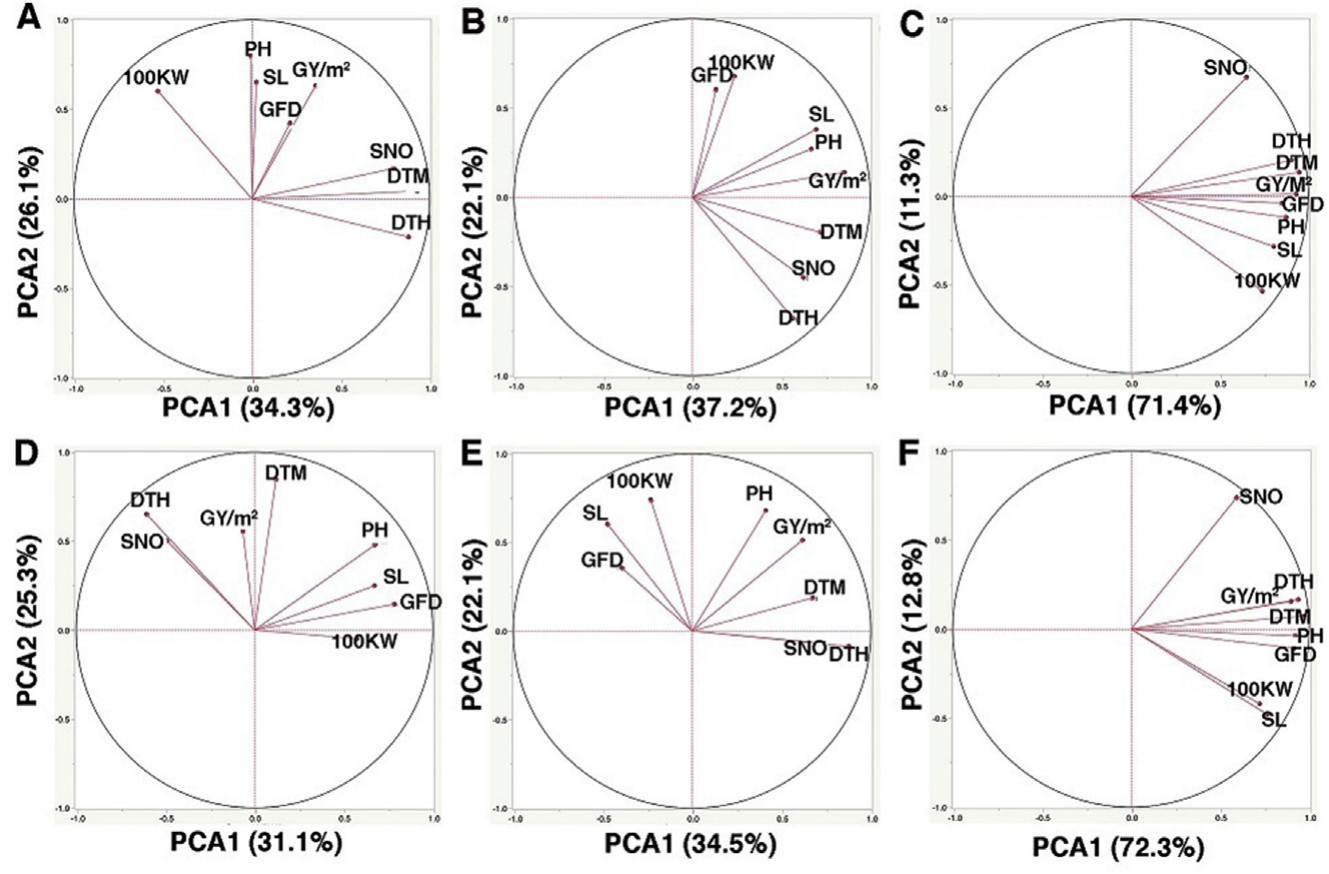
The Principal component analysis (PCA) of studied traits under both environmental conditions in S1 (A: normal environment, B: stress environment and C: both environment) and S2, (D: normal environment, E: stress environment and F: both environment).

The Principal Component Analysis was utilised when observing the multivariate dimension among the traits derived from a diverse genetic variance pool, the principal component analysis (PCA) (Figure 6) was utilised. The whole variation was aggregated by seven principal components. The first three components held 88.6% and 90.5% when combining the NE and SE data of S1 and S2, respectively (Table 9). There was a slight change in traits dimension according to the season (Figure 6-C, F) and it was noted that the SNO was not sufficiently stable across seasons (see the loading values and trait clusters in Table 10). Generally, by separating the NE from SE in each season, less variations were explained in S2, where the first three PCAs explained 77.4% and 74.4% of the variation in S1 versus 73.5% and 72.0% in S2, respectively (Table 9). Also, according to the correlation between the loading values and the PCAs for each trait, the traits were grouped into three clusters in S1 and only two clusters in S2. Although GY/m^2^, DTM, PH and SNO traits were clustered tightly under SE regardless of the impact of the season (Figure 6-B, E), this observation needs to be combined with the contribution of these traits in loading values and traits clustering (Table 10). Across seasons, the traits of PH and GY/m^2^ were the most stable traits and loaded the highest positive contribution under SE conditions while the least reliable traits were 100 KW and GFD. The rest of the traits came after PH and GY/m^2^.

**Table 9:** The corresponding eigenvalues, percent of each eigenvalue, and the accumulated percent to each treatment and seasonal variation. The eigenvalues were shaded in a gradient scale (from the dark to the faint) in descending order.

Treatment	Season	Number	Eigenvalue	Percent	Cum Percent
NE	S1	1	2.74	34.25	34.25
NE	S1	2	2.0899	26.124	60.374
NE	S1	3	1.3662	17.078	77.452
NE	S1	4	0.673	8.412	85.864
NE	S1	5	0.5202	6.502	92.366
NE	S1	6	0.414	5.175	97.541
NE	S1	7	0.1967	2.459	100
SE	S1	1	2.976	37.2	37.2
SE	S1	2	1.7702	22.128	59.328
SE	S1	3	1.2124	15.155	74.482
SE	S1	4	0.8874	11.092	85.575
SE	S1	5	0.528	6.6	92.174
SE	S1	6	0.4177	5.221	97.395
SE	S1	7	0.2084	2.605	100
NE	S2	1	2.4912	31.14	31.14
NE	S2	2	2.0235	25.293	56.433
NE	S2	3	1.3703	17.129	73.561
NE	S2	4	0.8112	10.139	83.701
NE	S2	5	0.6039	7.549	91.249
NE	S2	6	0.4757	5.946	97.195
NE	S2	7	0.2244	2.805	100
SE	S2	1	2.7578	34.472	34.472
SE	S2	2	1.8144	22.68	57.152
SE	S2	3	1.1911	14.889	72.041
SE	S2	4	0.9017	11.272	83.313
SE	S2	5	0.5829	7.287	90.6
SE	S2	6	0.5051	6.313	96.913
SE	S2	7	0.2469	3.087	100
	S1	1	5.7142	71.427	71.427
	S1	2	0.9025	11.281	82.708
	S1	3	0.4789	5.987	88.695
	S1	4	0.35	4.375	93.07
	S1	5	0.2612	3.265	96.335
	S1	6	0.1843	2.304	98.639
	S1	7	0.1089	1.361	100
	S2	1	5.7808	72.26	72.26
	S2	2	1.0223	12.779	85.039
	S2	3	0.4412	5.514	90.553
	S2	4	0.3195	3.994	94.547
	S2	5	0.1978	2.473	97.02
	S2	6	0.1378	1.722	98.742
	S2	7	0.1006	1.258	100

**Table 10:** The loading matrix shows the absolute loading values corresponding to the loading values for each component of the seven PCAs in association with clustering the traits. The coefficient of the clusters for each trait was listed according to each trait.

Treatment	Season	Trait	Prin1	Prin2	Prin3	Prin4	Prin5	Prin6	Prin7	cluster	Cluster1 Coefficient	Cluster2 Coefficient	Cluster 3 Coefficient
NE	S1	PH	-0.01	0.80	0.32	-0.28	-0.31	0.19	-0.21	3	0.00	0.00	0.68
NE	S1	SL	0.03	0.65	-0.56	0.37	-0.26	0.16	0.18	2	0.00	0.71	0.00
NE	S1	SNO	0.80	0.17	0.17	-0.47	-0.06	-0.08	0.28	1	0.53	0.00	0.00
NE	S1	GY/m2	0.36	0.63	-0.49	-0.05	0.16	-0.42	-0.14	2	0.00	0.71	0.00
NE	S1	100KW	-0.53	0.60	0.00	-0.15	0.53	0.23	0.08	3	0.00	0.00	0.51
NE	S1	DTH	0.88	-0.21	-0.29	0.00	0.13	0.26	-0.10	1	0.59	0.00	0.00
NE	S1	DTM	0.93	0.04	0.16	0.23	0.16	0.15	-0.06	1	0.61	0.00	0.00
NE	S1	GFD	0.21	0.42	0.76	0.41	0.07	-0.15	0.05	3	0.00	0.00	0.53
SE	S1	PH	0.67	0.27	-0.22	-0.29	-0.55	0.20	0.01	1	0.55	0.00	0.00
SE	S1	SL	0.70	0.38	-0.38	-0.06	0.12	-0.40	0.21	1	0.59	0.00	0.00
SE	S1	SNO	0.63	-0.45	0.30	-0.35	0.27	0.27	0.22	2	0.00	0.52	0.00
SE	S1	GY/m2	0.86	0.14	-0.01	-0.26	0.26	-0.03	-0.33	1	0.59	0.00	0.00
SE	S1	100KW	0.24	0.68	-0.28	0.48	0.22	0.36	0.04	3	0.00	0.00	0.71
SE	S1	DTH	0.57	-0.68	-0.22	0.40	-0.08	-0.02	-0.03	2	0.00	0.60	0.00
SE	S1	DTM	0.72	-0.20	0.45	0.46	-0.14	-0.08	-0.01	2	0.00	0.61	0.00
SE	S1	GFD	0.13	0.60	0.78	0.04	-0.06	-0.07	0.03	3	0.00	0.00	0.71
NE	S2	PH	0.68	0.48	-0.23	-0.06	-0.09	0.44	-0.23	1	0.56	0.00	
NE	S2	SL	0.67	0.25	0.43	-0.24	-0.43	0.00	0.25	1	0.52	0.00	
NE	S2	SNO	-0.49	0.50	0.07	0.63	-0.06	0.26	0.17	2	0.00	0.48	
NE	S2	GY/m2	-0.07	0.56	0.72	0.18	-0.08	-0.26	-0.25	2	0.00	0.43	
NE	S2	100KW	0.58	-0.05	0.54	0.01	0.59	0.14	0.09	1	0.40	0.00	
NE	S2	DTH	-0.61	0.65	-0.01	-0.43	0.13	0.05	0.05	2	0.00	0.58	
NE	S2	DTM	0.12	0.85	-0.40	-0.12	0.19	-0.22	0.08	2	0.00	0.50	
NE	S2	GFD	0.79	0.15	-0.40	0.35	0.05	-0.27	0.03	1	0.50	0.00	
SE	S2	PH	0.41	0.68	-0.16	-0.04	-0.30	-0.50	-0.07	1	0.36	0.00	
SE	S2	SL	-0.47	0.60	-0.17	-0.46	0.31	-0.03	0.27	2	0.00	0.61	
SE	S2	SNO	0.74	-0.07	-0.22	0.54	0.07	-0.04	0.32	1	0.41	0.00	
SE	S2	GY/m2	0.62	0.51	-0.13	0.16	0.49	0.11	-0.24	1	0.47	0.00	
SE	S2	100KW	-0.23	0.74	-0.18	0.21	-0.36	0.44	0.01	2	0.00	0.64	
SE	S2	DTH	0.88	-0.09	0.04	-0.42	-0.11	0.16	0.04	1	0.51	0.00	
SE	S2	DTM	0.68	0.19	0.67	-0.21	-0.06	0.10	0.07	1	0.47	0.00	
SE	S2	GFD	-0.39	0.36	0.77	0.33	0.08	-0.10	0.04	2	0.00	0.46	
	S1	PH	0.88	-0.12	0.08	0.33	-0.15	-0.28	-0.04	1	0.37		
	S1	SL	0.81	-0.28	0.44	-0.14	-0.16	0.09	0.15	1	0.34		
	S1	SNO	0.65	0.68	0.13	0.25	0.13	0.11	0.09	1	0.27		
	S1	GY/m2	0.93	0.01	0.19	-0.05	0.04	0.12	-0.27	1	0.39		
	S1	100KW	0.74	-0.54	-0.13	0.14	0.34	0.06	0.05	1	0.31		
	S1	DTH	0.90	0.20	-0.07	-0.30	0.11	-0.17	0.03	1	0.38		
	S1	DTM	0.95	0.14	-0.19	-0.20	-0.01	-0.06	0.03	1	0.40		
	S1	GFD	0.85	-0.04	-0.41	0.07	-0.26	0.18	0.02	1	0.36		
	S2	PH	0.92	-0.04	-0.08	-0.06	0.29	-0.21	-0.09	1	0.40	0.00	
	S2	SL	0.77	-0.48	-0.08	0.39	0.10	0.09	0.09	1	0.34	0.00	
	S2	SNO	0.59	0.74	0.23	0.09	0.16	0.12	0.06	2	0.00	1.00	
	S2	GY/m2	0.90	0.16	0.14	0.24	-0.23	-0.07	-0.17	1	0.38	0.00	
	S2	100KW	0.72	-0.42	0.51	-0.19	-0.02	0.00	0.05	1	0.32	0.00	
	S2	DTH	0.94	0.17	-0.16	-0.07	-0.14	-0.11	0.17	1	0.40	0.00	
	S2	DTM	0.97	0.07	-0.18	-0.12	-0.09	0.01	0.06	1	0.41	0.00	
	S2	GFD	0.92	-0.11	-0.19	-0.21	0.01	0.23	-0.13	1	0.40	0.00	

**Figure 7:**
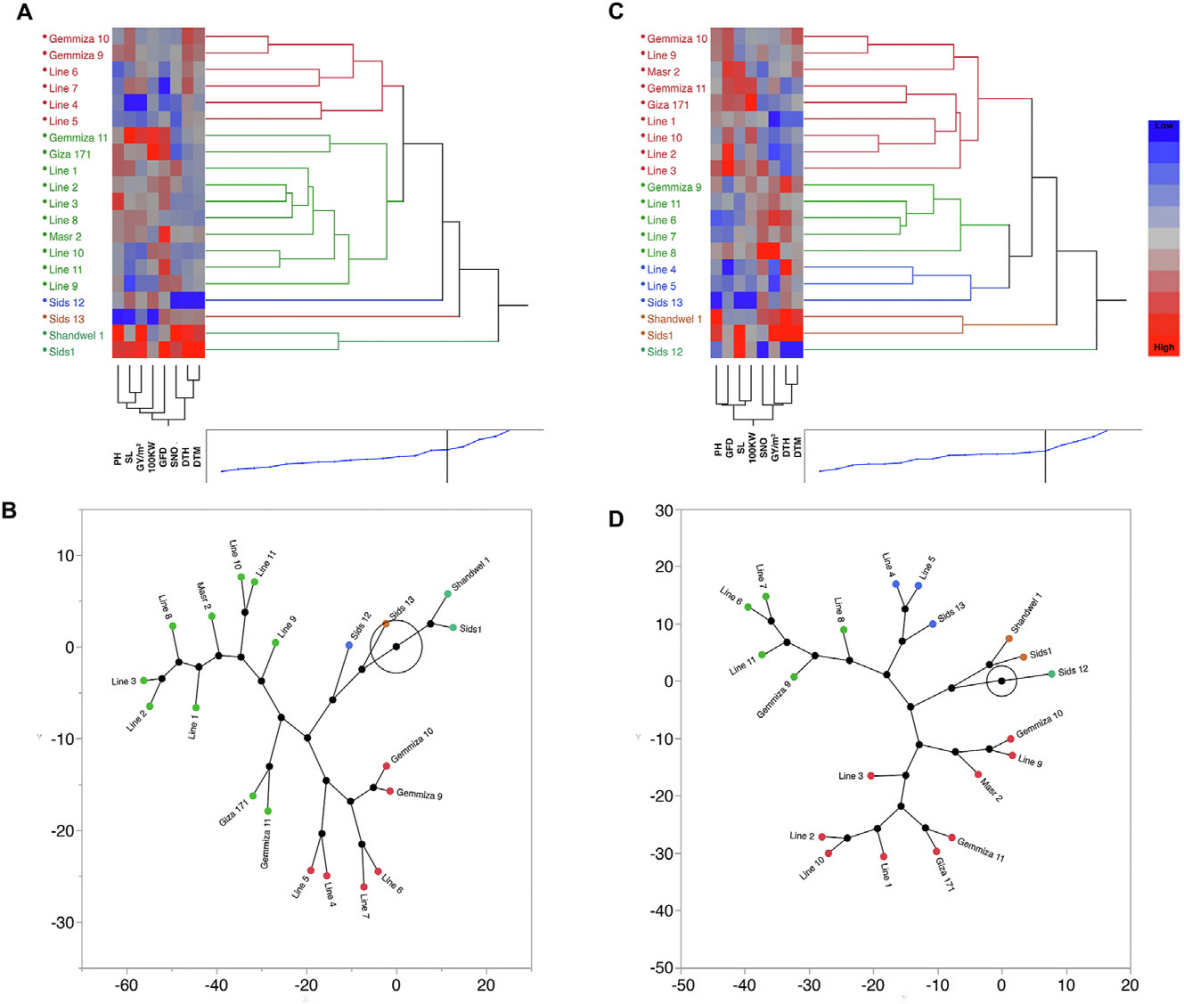
Two-way hierarchical clustering heatmaps of the 20 genotypes using all studied traits under both environmental conditions supported by a constellation plot in S1 (A and B) and S2 (C and D). Genotypes are clustered into 5 colored clusters based on K-means cluster approach (Red, Green, Blue, Brown, and Turquoise). In the constellation plot, the genotypes were arranged as endpoints and each cluster represents a new point, lines refer to distance between clusters.

Moreover, to overview the traits and genotypes dimensions, we used the two-way hierarchy clustering approach, which built based on all the studied genotypes and traits per season (Figure 7A, C). The constellation plots (Figure 7 B, D) corresponding to each season helped visualize the genotypes as endpoints and divide them into five clusters in which the lines between the endpoints represented the distance between clusters. In both seasons, we observed that Masr2, Giza 171, Gemmiza 11, Line 1, Line 2, Line 3, Line 9 and Line 10 constructed a consistent cluster under S1 (green cluster) and S2 (Red cluster). On the other hand, Gemmiza 10 was integrated in the same cluster in S2 to substitute Line 8 which clustered separately. Some genotypes showed consistency in sharing the same group with other certain genotypes across seasons, such as Shandwel1 and Sids1, Lin6 and Line7, and Gemmiza11 and Giz171. Sids12 was clustered separately in both seasons. In both seasons, Gemmiza9, Line4, Line5, Line6, Line7, and Sids13 genotypes were clustered together, and in the meantime scored poor performance across the studied traits.

## 7 Conclusion

Heat tolerance is a quantitative trait that is promoted by several components. Investigating the correlation between phenotypic traits targets the prediction of reliable traits for genotype selection. This approach is necessary to facilitate wheat breeding programs and understand mechanisms for heat tolerance. In our study, we concluded that heat stress that is applied by late sowing procedure has negatively affected all studied traits, and slightly influenced pollen grain viability and germinability of producing grains. The grain yield/m^2^ was found as the most reliable trait for genotype selection. Once established, the phenotypic traits, SL and SNO, may be considered as a selection criterion for heat tolerance. The Egyptian cultivars Masr2, Giza171 and Sids1 have a plastic response to heat stress. Meanwhile, Gemmiza9, Gemmiza10, and Sids13 started to show mal-adaptive performance under stress. It was found that Line4, Line5, Line6, Line7, Line8, and Line11 are unappreciated lines under local heat conditions.

## Declarations

### Author contribution statement

All authors: conceived and designed the experiments; Shenoda, J. E.; Marwa, N. M. E. Sanad; Aida, A. Rizkalla :and Rania, T. Ali: performed the experiments; Shenoda, J. E.; Marwa, N. M. E. Sanad; Aida, A. Rizkalla analyzed and interpreted the data; All authors: contributed reagents, materials, analysis tools or data; Shenoda, J. E.; Marwa, N. M. E. Sanad; Aida, A. Rizkalla Wrote the paper.

### Funding statement

This work was supported by the 10.13039/100007787National Research Center (NRC), Egypt.

### Data availability statement

Data included in article/supplementary material/referenced in article.

### Declaration of interests statement

The authors declare no conflict of interest.

### Additional information

No additional information is available for this paper.
